# Occurrence and Behavior of Methylsiloxanes in Urban Environment in Four Cities of China

**DOI:** 10.3390/ijerph192113869

**Published:** 2022-10-25

**Authors:** Yao Jiang, Junyu Guo, Ying Zhou, Boya Zhang, Jianbo Zhang

**Affiliations:** 1 College of Life and Environmental Sciences, Minzu University of China, Beijing 100081, China; 2 State Key Joint Laboratory for Environmental Simulation and Pollution Control, College of Environmental Sciences and Engineering, Peking University, Beijing 100871, China; 3 Department of Epidemiology, University of Michigan, Ann Arbor, MI 48103, USA

**Keywords:** methylsiloxane, multi-media environment, environmental behavior, EQC model, monitoring

## Abstract

Methylsiloxanes (MSs), used in industrial production and personal care products, are released in various environmental media. In this study, we combined monitoring and modeling to investigate the occurrence and behavior of MSs in the urban environment in China. MSs were widely found in the air, water, soil and sediment of four cities in China. The concentrations of MSs in all four environmental media of Zhangjiagang were higher than those in the other three cities (Beijing, Kunming and Lijiang), indicating that the siloxane production plant had a significant impact on the pollution level of MSs in the surrounding environment. The samples with high MS concentrations were all from the sample sites near the outlet of the WWTPs, which showed that the effluent of the WWTPs was the main source of MS pollution in the surrounding environment. The modeling results of the EQC level III model showed that D4 discharged into the environment was mainly distributed in the air, while D5 and D6 were mainly distributed in the sediment. CMSs (D4–D6) discharged into various environmental media could exist in the urban environment for a long time with low temperatures in cities. When the temperature was 0 °C, the residence time of D5 and D6 could be 68.1 days and 243 days in the whole environmental system in Beijing. This study illustrates the importance of CMSs (D4–D6) in low-temperature environments and the potential environmental risks that they may pose.

## 1. Introduction

Methylsiloxanes (MSs) have been widely used in industrial production and consumer products for decades, and are a group of substances consisting of cyclic MSs (CMSs) and linear MSs (LMSs) [1,2,3]. MSs are mass-produced worldwide, with an annual production of approximately 8,000,000–10,000,000 tons, of which China is the largest manufacturer and consumer of these chemicals [4]. Because of their broad usage, octamethylcyclotetrasiloxane (D4), decamethylcyclopentasiloxane (D5) and dodecamethylcyclohexasiloxane (D6) have been classified as high-production-volume chemical substances by the Organization for Economic Co-operation and Development and the United States Environmental Protection Agency [5,6]. Because of their possible hazardous and toxic effects on the environment and humans, CMSs have been receiving increasing attention regarding their risks to the environment and have attracted the attention of regulators [7,8]. D4 and D5 have been listed as very persistent and bioaccumulative (vPvB) substances by the European Chemicals Agency [7], and these two cyclic compounds are currently being considered as emerging persistent organic pollutants (POPs) for regulation [9].

Due to their mass manufacture and extensive applications, MSs have been detected in various environmental matrices, including air [10,11,12,13], water [14,15,16,17], soil [18,19], sediment [15,20,21], and biota [22,23,24,25]. Canada and the UK have assessed the environmental risk for CMSs (D4–D6), and the results showed that MSs could have potential adverse effects on the environment [26,27,28,29]. Experimental studies have been reported that CMSs have toxic effects on the immune, nervous, endocrine, and reproductive systems; cyclic compounds can cause hormone abnormalities, organ damage, and reproductive failure [30,31,32,33]. MSs have been used extensively as additives in personal care products (PCPs) and household products, meaning that these compounds exist widely in the urban environment, especially in big cities [10,16]; due to the large population and high consumption level, this may lead to a high contaminant level of MSs in the urban environment. Because of their potential for persistence and toxicity, they may have lasting negative effects on human health [7,8]. An understanding of the environmental behavior of MSs will help to develop strategies for the management of such risks. However, there are few studies conducted so far focused on the environmental behavior of MSs in Chinese cities. Thus, it is important to understand the occurrence and environmental behavior of MSs in Chinese cities, and how environmental conditions influence the behavior of MSs.

The objectives of the present study were (1) to investigate the occurrence and distribution characteristics of MSs in the urban environment in China; (2) to simulate the exchange and transformation processes of MSs between air, water, soil and sediment in the urban environment with the fugacity-based multimedia model; (3) to understand the behavior of MSs in the urban environment in China by combining monitoring and modeling results. To our knowledge, studies on the environmental behavior of MSs in Chinese cities are still lacking. This study will be favorable for understanding the environmental fate of MSs in Chinese cities, and will provide useful data for the development of MS risk management strategies.

## 2. Materials and Methods

### 2.1. Sample Collection

In 2017, air (n = 156), water (n = 30), soil (n = 60), and sediment (n = 30) samples were collected from four cities in China: Beijing, Kunming, Zhangjiagang and Lijiang (Figure S1). Zhangjiagang has the largest siloxane production plant in China, but there are no siloxane production plants in Beijing, Kunming and Lijiang. ISOLUTE ENV+ 200 mg cartridges (Biotage AB, Uppsala, Sweden), polytetrafluoroethylene (PTFE) bottles, steel syringes, and BEEKER sediment samplers were used to collected air, water, soil, and sediment samples, respectively, as previously described [12,16]. Air samples were collected in the urban and suburban sites for each city. Two parallel air samples were collected at each sample site, and each sample was collected for 24 h with sampling rates of 2 L/min. Two water and two sediment samples were collected from the main natural rivers in each city. Soil samples were collected near each river sampling site, and two parallel soil samples were collected at each sampling site. The sample collection procedures are described in detail in the Supporting Information (SI), Section S1.2.

### 2.2. Analytical Methods

In this study, the extraction methods and quantitative analysis were performed according to previously published methods [12,16]. The detailed procedures are provided in SI Section S1.3. Briefly, each sample was spiked with internal standards followed by an extraction procedure. The extract was concentrated to 1 mL by a gentle stream of nitrogen and subjected to gas chromatography–mass spectrometry (GC-MS) analysis. The same preparation methods were performed in corresponding field blanks from the study sites. All samples were pretreated and analyzed within 4 days of the sample collection.

### 2.3. Quality Assurance and Quality Control

Due to the extensive use of MSs in many consumer products, precautions were carried out to prevent contamination during sample collection and the analytical procedures. Silicon-based materials and products containing siloxanes were prohibited. All glassware was treated at 300 °C for 4 h and then cleaned with n-hexane before use. Each set of samples was analyzed in accordance with previously described quality assurance protocols [12]. Double procedural blanks were used to monitor contamination during sample preparation. Double reagent blanks were used to check for background contamination and carry-over. Field blanks were used to ensure that no contamination occurred during sampling or transportation. Blank cartridges, ultrapure water, blank soil, and blank sediment were used as field blanks for the air, water, soil, and sediment samples, respectively. The limit of detection (LOD) and limit of quantitation (LOQ) were set at a signal-to-noise ratio of three and ten in the sample extracts, respectively. The LOQ of the MSs D4–D6 and L5–L16 were 0.19–0.37 ng/m^3^, 9.0–18.3 ng/L, 0.5–0.8 ng/g, and 0.6–0.8 ng/g for air, water, soil, and sediment, respectively. Field blank levels were all below the limit of quantitation for all LMSs. CMSs (D4–D6) were detected in blanks of air (0.08–0.11 ng/m^3^), water (1.0–2.1 ng/L), soil (0.2–0.6 ng/g dw), and sediment (0.2–0.6 ng/g dw), respectively. Blank values were subtracted from the sample concentrations. The relative standard deviations (%) of MS concentrations in parallel samples (air, water, soil, and sediment) were all < 15% (details in SI Section S1.4).

### 2.4. Modeling Assessment

In this study, the behavior and fate of CMSs (D4–D6) in urban environment were simulated using the EQuilibrium Criterion (EQC) model. EQC Level III is a steady-state non-equilibrium model that simulates exchange and transformation processes between air, water, soil, and sediment [34,35]. The EQC Level III model requires input of the physicochemical property parameters and emission data for chemicals. The physicochemical property parameters of CMSs (D4–D6) used for model input are shown in Table S3. Because of the temperature dependence of chemical properties, the behavior and fate of chemicals in multi-environmental media could be affected. Thus, the model was run for these four temperature scenarios (0 °C, 15 °C, 25 °C and 35 °C). In the EQC model of this study, the value of the air–water partition coefficient (K_AW_), the octanol–water partition coefficient (K_OW_) and the organic carbon–water partition coefficient (K_OC_) were defined. All principal partition coefficients (K_AW_, K_OW_ and K_OC_) were temperature-adjusted using values for the energies of phase change in the model (SI Section S1.5) [36,37]. The direct emissions of CMSs (D4–D6) are estimated based on the amount of MSs in personal care products used per capita [36]. The previous studies indicated that 90% of CMSs (D4–D6) used for personal care products by urban residents were released into the atmosphere and 10% were discharged into sewage water [27,28,29]. According to a previous study on the discharge data of CMSs (D4–D6) from wastewater treatment plants (WWTPs) in Beijing [38], the per capita discharge of CMSs (D4–D6) could be calculated. The rates of CMS (D4–D6) emission into the atmosphere and water were calculated based on the resident population of Beijing and the removal efficiency of WWTPs. In 2017, the population in Beijing was 21,700,000 [39]. Because the surplus sludge of the WWTPs will eventually be landfilled or used for composting treatment, the rate of CMS (D4–D6) discharge into the soil can be estimated based on the daily discharge amount and CMS levels of surplus sludge. The emission rates of CMSs (D4–D6) for atmosphere, water, and soil are shown in Table S4.

## 3. Results and Discussion

### 3.1. Concentrations of MSs in Four Cities

#### 3.1.1. Air

Of the 156 air samples collected in the urban and suburban sites from four cities, three CMS (D4–D6) were detected in all air samples. The mean concentrations of CMSs (D4–D6) in the urban air of Beijing, Kunming, Zhangjiagang and Lijiang were 172 ng/m^3^, 136 ng/m^3^, 215 ng/m^3^, and 69.8 ng/m^3^, respectively, and in suburban air, they were 88.0 ng/m^3^, 71.2 ng/m^3^, 112 ng/m^3^, and 24.6 ng/m^3^ (Table 1). The result showed that the concentrations of CMSs (D4–D6) in the urban air were two to three times higher than those in the suburban air. The possible reason is that one of the major sources of MSs in urban air is the use of personal care products, and most people in cities live in the urban area [40]. Concentrations of CMSs (D4–D6) in the air of the four cities were higher than those in other rural areas (United States and Sweden) and a polar area (Arctic) [41,42,43]. In all air samples, D5 exhibited the highest measured concentration among the three CMSs (D4–D6). The concentrations of CMSs (D4–D6) in the air of Zhangjiagang were higher than those in the other three cities. The possible reason is that Zhangjiagang has a lot of siloxane production plants, including one of the largest siloxane factories in the world. Due to the emission in the production process, the environmental pollution of MSs in Zhangjiagang was more serious than other cities.

The mean concentrations of CMSs (D4–D6) and LMSs (L5–L8) detected in the air around siloxane production plant in Zhangjiagang were 335 μg/m^3^ and 0.51 μg/m^3^, respectively (Table 1). Twelve LMSs (L5–L16) were not detected in the air 500 m upwind of the siloxane production plant, and the mean concentration of CMSs (D4–D6) was 7.5 μg/m^3^, while in the air 500 m downwind of the siloxane production plant, the mean concentrations of CMSs (D4–D6) and LMSs (L5–L8) were 64.6 μg/m^3^ and 56.2 ng/m^3^, respectively. The result indicated that a large number of MSs could be discharged into the surrounding environment during the production process. During the sampling period, the wind direction of Zhangjiagang is mainly in the southeast, and the plant is located in the northwest corner of the city; thus, the MS emission from the siloxane production plant has little effect on the atmospheric environment of the city. However, the wind direction in this area is mainly northerly in autumn and winter; the large amount of MSs emitted by the production plant may cause critical air pollution in this city. In addition, the mean concentrations of CMSs (D4–D6) and LMSs (L5–L8) at 500 m downwind of the siloxane production plant were 2.1 and 9.1 times lower than those around the plant, respectively, and only CMSs (D4–D6) were detected in the air 500 m upwind of the plant. The reason may be due to the high volatility of CMSs, compared with LMSs, as cyclic compounds can migrate long distances in the atmospheric environment [40].

#### 3.1.2. Water

The concentrations of CMSs (D4–D6) detected in water from Beijing, Kunming and Lijiang were 17.1–257 ng/L, 12.6–189 ng/L, and <LOD–65.1 ng/L, respectively (Table 2). In all water samples, the mean concentration of CMSs (D4–D6) in Beijing (49.0 ng/L) was higher than Kunming (33.5 ng/L) and Lijinag (28.5 ng/L). The samples with high MS concentrations were all from the sample sites near the outlet of the WWTPs, and the detection rates of CMSs (D4–D6) were 100%. Because the population of Beijing is much larger than that of Kunming, and the total use of personal care products is relatively high, the total amount of MSs discharged into sewage is relatively large. The concentrations of CMSs (D4–D6) in water samples near the outlet of the WWTPs were one order of magnitude higher than other water samples, indicating that the effluent of the WWTPs was the main source of MS pollution in natural water. LMSs were not detected in all water samples in four cities, except for one sampling site in Zhangjiagang, with concentrations of 59.6 ng/L for LMSs (L3–L5). Among the water samples in Zhangjiagang, the concentrations of the MSs in the natural water were much higher than those in northern Europe and England [14,44]. Relatively high concentrations of CMSs (D4–D6) were detected in the sampling sites near the siloxane production plant, with the mean concentration of 512 ng/L, which was two to four times higher than those in the sampling sites at the outlet of the WWTPs in Beijing and Kunming. This sampling site is very close to the siloxane production plant (the straight-line distance is less than 300 m); there are many textile factories around the sampling point, and MSs are widely used in finishing agents and softeners [45]. Thus, the production activities of the textile factory could cause MS pollution to the surrounding water. In addition, the mean concentration of CMSs (D4–D6) was 79.9 ng/L in other water samples in Zhangjiagang.

#### 3.1.3. Soil and Sediment

The mean concentrations of MSs in soil and sediment from four cities are shown in Table 3. MSs were not detected in all soil samples in three cities (Beijing, Kunming and Lijiang), except for sampling sites at the outlet of the WWTPs, with mean concentrations of 1.5–2.0 ng/g dw for CMSs (D4–D6). In Zhangjiagang, the mean concentration of CMSs (D4–D6) in soil near the siloxane production plant (212 ng/g dw) was two orders of magnitude higher than those in other sampling sites; meanwhile, these concentrations were one to two orders of magnitude higher than those in Spain [18,46]. LMSs (L5–L11) were only detected in soil near the siloxane production plant, with concentrations of 102 ng/g dw. 

In Beijing and Kunming, the mean concentrations of CMSs (D4–D6) and LMSs (L5–L11) in sediment near the outlet of the WWTPs were 2220–3350 ng/g dw and 764–1012 ng/g dw, respectively, which were one order of magnitude higher than those in the other sediment samples (CMSs: 189–782 ng/g dw; LMSs: 85.2–223 ng/g dw). Thus, the effluent of WWTPs is one of the main pollution sources of MSs in the urban soil and sediment environment. The CMSs (D4–D6) and LMSs (L5–L11) in sediment samples from Lijiang were only detected in the downtown sampling sites, with mean concentrations of 85.4 ng/g dw and 36.5 ng/g dw for CMSs (D4–D6) and LMSs (L5–L11), respectively. The highest concentration of MSs in sediment was detected at the sampling site near the siloxane production plant, with mean concentrations of 4261 ng/g dw and 1694 ng/g dw for CMSs (D4–D6) and LMSs (L5–L11), respectively, which were one to two orders of magnitude higher than those in the United Kingdom and Canada [47,48,49]. In other sampling sites of Zhangjiagang, the mean concentrations of CMSs (D4–D6) and LMSs (L5–L11) in sediment were 166–782 ng/g dw and 95.5–283 ng/g dw, respectively, which were one to two orders of magnitude lower than those in sampling site near the siloxane production plant.

### 3.2. Model Simulation and Evaluation

Four different temperature scenarios (0 °C, 15 °C, 25 °C and 35 °C) were used to simulate the fraction of the total mass of CMSs (D4–D6) in the urban environment which reside in air, water, soil, and sediment, respectively (Table 4). For the four environmental media, the vast majority of D4 (91.2–99.5%) was predicted to be in the air, indicating that D4 is discharged mostly into the environment in air, while most D5 and D6 were predicted to be in sediment, accounting for 87.2–88.1% and 91.3–94.5%, respectively. The fraction of the total mass of three CMSs (D4–D6) in water and soil were all less than 5%. The possible reason is that the vapor pressure of D4 is much higher than that of D5 and D6, and the K_OW_ and K_OC_ values of D5 and D6 are larger than that of D4 [27,28,29]. Meanwhile, the amounts of three CMSs (D4–D6) discharged into the air and water were far greater than that of soil in the emission scenarios of the model. Thus, D4 discharging into the water tends to volatilize into the air, while D5 and D6 emitting into the air tend to deposit into water and subsequently adsorb into sediment. The modeling results could explain that the concentrations of CMSs (D4–D6) in sediment were higher than those in soil at the same sampling sites in Beijing and Kunming.

In order to verify the modeling results of the EQC level III model, the fraction of the total mass of CMSs (D4–D6) in each environmental media was estimated by using the mean concentrations of CMSs (D4–D6) in each environmental media in Beijing detected in this study, combined with the actual environmental parameters in Beijing. The environmental parameters refer to the first national geographic survey bulletin of Beijing (Table S5). Because the sample collection was carried out in June 2017 (with the mean temperature of 25.1 °C), the estimated results were compared with the modeling results under the 25 °C scenario. 

**Figure 1:**
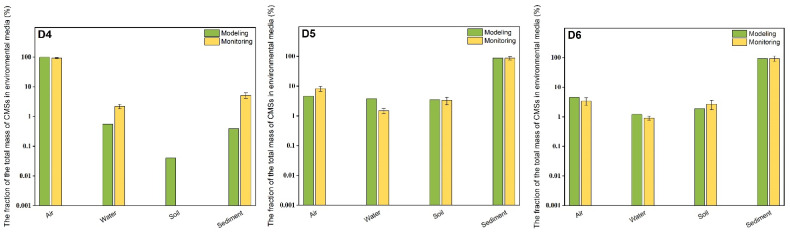
The actual value (%) and simulated value (%) of the fraction of the total mass of CMSs (D4–D6) in each environmental media in Beijing.

The actual value (%) of the fraction of the total mass of CMSs (D4–D6) in each environmental media in Beijing is shown in Table S6. The actual proportions of D5 in air, water, soil, and sediment are 8.2%, 1.5%, 3.3% and 87.0%, respectively; for D6, the corresponding actual proportions are 3.4%, 0.91%, 2.7% and 92.9% (Figure 1). These results were close to the modeling results at 25 °C, indicating that the EQC level III model could accurately simulate the behavior of D5 and D6 in four different environment media in Beijing. However, the actual proportions of D4 in water and sediment were 2.2% and 5.2%, both of which were one order of magnitude higher than the modeling values; the actual proportion of D4 in air was 92.6%, which was lower than the modeling value. The possible reason is that among the sampling sites of water and sediment in Beijing, three sites are located near the outlet of WWTPs. D4 has just been discharged into the water and has not yet been volatilized into the air, which leads to a high proportion of D4 in water and sediment.

Under the four different temperature scenarios (0 °C, 15 °C, 25 °C and 35 °C), the total residence time, advection residence time, and reaction residence time of CMSs (D4–D6) in the whole environmental system are shown in Table 5. The reaction residence time of CMSs (D4–D6) decreased with the increase in temperature, indicating that the increase in temperature could lead to the acceleration of the chemical reaction rate. Under the three different temperature scenarios (0 °C, 15 °C, and 25 °C), the advection residence times of D4, D5 and D6 were less than the reaction residence times, indicating that advection transport played a major role in the fate of the three cyclic compounds in this environmental system. However, under the 35 °C scenario, the reaction residence time of D4, D5 and D6 was less than the advection residence time, indicating that chemical reaction played a major role in the fate of the three CMSs (D4–D6). In this environmental system, the total residence time of D4, D5 and D6 decreased with the increase in temperature. When simulated at 0 °C scenario, the residence time of D5 and D6 in the whole environmental system could be over 68.1 days and 243 days. A previous study on the fate of MSs in a Chinese lake showed that cVMS in the lake was mainly distributed in the sediment and could persist for several years, especially for D5 and D6 [16]. Thus, these results showed that CMSs (D4–D6) discharged into various environmental media could exist in the urban environment for a long time, especially when the mean temperature was lower than 0 °C in winter in Beijing. Meanwhile, due to the continuing emissions of MSs, D5 and D6 could accumulate and lead to high MSs pollution in this area. In 2018, the incorporation of D4 and D5 in “wash-off” PCPs was restricted by the European Chemicals Agency [50], and D4 and D5 were banned in wash-off cosmetic products in Europe in Jan 2021 [51]. Given that most MSs are marketed in China worldwide, a restriction regulation on the use of MSs should be introduced in China. 

## 4. Conclusions

In this study, the concentrations of MSs in the urban environment (air, water, soil, and sediment) in China were detected. MSs were widely found in the air, water, soil and sediment of the four cities in China, indicating that personal care products used by urban residents could discharge a large amount of MSs into the environment, which is one of the important sources of MS pollution in urban areas. The siloxane production plant had a significant impact on the pollution level of MSs in the surrounding environment, which could lead to high exposure risk of MSs for residents around the siloxane production plant. The behavior of MSs in the urban environment was assessed by the fugacity-based multimedia model. The modeling results of the EQC level III model showed that D4 discharged into the environment was dominantly distributed in the air (91.2–99.5%), while D5 (87.2–88.1%) and D6 (91.3–94.5%) were mainly distributed in the sediment. Compared with the monitoring results in Beijing, the EQC level III model could accurately simulate the behavior and fate of D5 and D6 in the environment of Beijing. The modeling results showed that a lower temperature was conducive to the residence of CMSs (D4–D6) in the environmental system, especially for D5 and D6. When the temperature was lower than 0 °C, the residence time of D5 and D6 could be 68.1 days and 243 days in the whole environmental system in Beijing. Therefore, cities with low temperatures should be receive more attention in future studies on the environmental behavior of MSs and exposure risks for residents.

## Figures and Tables

**Table 1:** The mean concentrations (ng/m^3^) of methylsiloxanes in air samples from four cities.

Site	D4	D5	D6	ΣL5–L8
Beijing (urban)	66.2	69.1	36.2	<LOD ^1^
Beijing (suburban)	40.5	35.6	11.9	<LOD
Kunming (urban)	52.6	55.1	28.5	<LOD
Kunming (suburban)	32.9	30.6	7.7	<LOD
Lijiang (urban)	29.4	33.5	6.9	<LOD
Lijiang (suburban)	12.3	11.2	1.1	<LOD
Zhangjiagang (urban)	75.6	83.1	55.9	<LOD
Zhangjiagang (suburban)	45.1	47.2	19.3	<LOD
Siloxane production plan (around)	1.3 × 10^5^	1.7 × 10^5^	3.6 × 10^4^	5.1 × 10^2^
Siloxane production plan (downwind)	3.3 × 10^4^	2.5 × 10^4^	6.6 × 10^3^	56.2
Siloxane production plan (upwind)	2.7 × 10^3^	4.1 × 10^3^	7.1 × 10^2^	<LOD

^1^ <LOD = levels lower than LOD.

**Table 2:** The mean concentrations (ng/L) of methylsiloxanes in water samples from four cities.

City	Site	D4	D5	D6	ΣL3–L5
Beijing	River a (1)	<LOD ^1^	17.1	<LOD	<LOD
	River a (2)	<LOD	15.6	<LOD	<LOD
	River b (1)	<LOD	12.8	<LOD	<LOD
	River b (2)	<LOD	13.1	<LOD	<LOD
	River c (1)	<LOD	19.9	18.8	<LOD
	River c (2)	<LOD	20.5	19.5	<LOD
	River d (1)	20.2	132	105	<LOD
	River d (2)	10.8	62.3	38.5	<LOD
	River e (1)	15.3	113	69.2	<LOD
	River e (2)	9.5	44.3	30.3	<LOD
	River f (1)	16.5	122	88.1	<LOD
	River f (2)	10.2	52.7	45.6	<LOD
Kunming	River g (1)	<LOD	13.3	<LOD	<LOD
	River g (2)	<LOD	12.6	<LOD	<LOD
	River h (1)	15.3	108	65.3	<LOD
	River h (2)	<LOD	35.3	23.1	<LOD
	River j (1)	12.2	85.3	51.5	<LOD
	River j (2)	<LOD	29.1	20.6	<LOD
Lijiang	River k (1)	<LOD	25.2	19.6	<LOD
	River k (2)	10.2	31.5	23.4	<LOD
	River m (1)	<LOD	<LOD	<LOD	<LOD
	River m (2)	<LOD	<LOD	<LOD	<LOD
	River n (1)	<LOD	<LOD	<LOD	<LOD
	River n (2)	<LOD	<LOD	<LOD	<LOD
Zhangjiagang	River p (1)	<LOD	25.1	21.2	<LOD
	River p (2)	<LOD	21.7	19.1	<LOD
	River q (1)	10.6	56.9	36.1	<LOD
	River q (2)	9.9	52.2	34.5	<LOD
	River r (1)	145	191	176	59.6
	River r (2)	11.5	61.3	39.2	<LOD

^1^ <LOD = levels lower than LOD.

**Table 3:** The mean concentrations (ng/g dw) of methylsiloxanes in soil and sediment samples from four cities.

City	Site	Soil				Sediment			
		D4	D5	D6	ΣL5–L11	D4	D5	D6	ΣL5–L11
Beijing	River a (1)	<LOD ^1^	17.1	<LOD	<LOD	0.88	102	136	115
	River a (2)	<LOD	<LOD	<LOD	<LOD	0.81	91.8	128	109
	River b (1)	<LOD	<LOD	<LOD	<LOD	0.65	78.1	98.5	101
	River b (2)	<LOD	<LOD	<LOD	<LOD	0.71	85.7	107	108
	River c (1)	<LOD	<LOD	<LOD	<LOD	0.96	156	192	150
	River c (2)	<LOD	<LOD	<LOD	<LOD	0.98	172	206	166
	River d (1)	<LOD	1.10	0.94	<LOD	8.50	1582	1759	1012
	River d (2)	<LOD	<LOD	<LOD	<LOD	1.20	355	426	223
	River e (1)	<LOD	0.82	0.73	<LOD	6.60	1195	1429	764
	River e (2)	<LOD	<LOD	<LOD	<LOD	1.10	262	314	197
	River f (1)	<LOD	0.85	0.78	<LOD	7.10	1321	1462	832
	River f (2)	<LOD	<LOD	<LOD	<LOD	1.10	325	401	210
Kunming	River g (1)	<LOD	<LOD	<LOD	<LOD	0.89	87.1	106	106
	River g (2)	<LOD	<LOD	<LOD	<LOD	0.82	85.2	103	85.2
	River h (1)	<LOD	1.10	0.89	<LOD	6.90	1039	1250	684
	River h (2)	<LOD	<LOD	<LOD	<LOD	1.10	247	295	166
	River j (1)	<LOD	0.81	0.72	<LOD	5.70	1005	1209	636
	River j (2)	<LOD	<LOD	<LOD	<LOD	0.97	203	258	141
Lijiang	River k (1)	<LOD	<LOD	<LOD	<LOD	<LOD	<LOD	<LOD	<LOD
	River k (2)	<LOD	<LOD	<LOD	<LOD	<LOD	<LOD	<LOD	<LOD
	River m (1)	<LOD	<LOD	<LOD	<LOD	<LOD	<LOD	<LOD	<LOD
	River m (2)	<LOD	<LOD	<LOD	<LOD	<LOD	<LOD	<LOD	<LOD
	River n (1)	<LOD	<LOD	<LOD	<LOD	0.64	38.2	51.3	37.9
	River n (2)	<LOD	<LOD	<LOD	<LOD	0.62	33.6	46.5	35.1
Zhangjiagang	River p (1)	<LOD	<LOD	<LOD	<LOD	0.69	74.2	91.6	95.5
	River p (2)	<LOD	<LOD	<LOD	<LOD	0.75	85.1	104	99.3
	River q (1)	<LOD	<LOD	<LOD	<LOD	0.91	106	135	117
	River q (2)	<LOD	<LOD	<LOD	<LOD	0.83	101	127	108
	River r (1)	29.6	96.7	85.3	102	55.3	1965	2241	1694
	River r (2)	<LOD	<LOD	0.76	<LOD	1.20	366	415	283

^1^ <LOD = levels lower than LOD.

**Table 4:** Predicted fractions of the total mass of CMSs (D4–D6) from modelling the four different environmental media of Beijing.

Chemical	Temperature (°C)	Air (%)	Water (%)	Soil (%)	Sediment (%)
D4	0	92.0	2.8	0.02	5.2
	15	97.7	0.88	0.03	1.4
	25	99.2	0.40	0.04	0.41
	35	99.6	0.20	0.09	0.09
D5	0	5.2	4.8	3.0	87.0
	15	5.7	4.3	4.0	86.0
	25	8.3	4.3	6.3	81.2
	35	17.6	4.5	12.0	65.9
D6	0	1.4	1.3	3.9	93.5
	15	5.2	1.2	3.0	90.6
	25	6.2	1.3	2.6	90.0
	35	7.5	1.6	2.9	88.1

**Table 5:** Residence time (d) of CMSs (D4–D6) in the environmental system of Beijing.

Chemical	Temperature (°C)	Total Residence Time	Advection Residence Time	Reaction Residence Time
D4	0	4.4	4.5	134
	15	3.5	4.3	20.6
	25	2.5	4.2	6.3
	35	1.6	4.2	2.5
D5	0	68.1	70.8	1773
	15	51.2	66.3	225
	25	20.4	47.1	48.7
	35	6.9	22.9	9.9
D6	0	243	249	9738
	15	61.8	76.0	329
	25	34.4	64.0	74.5
	35	16.0	53.1	22.8

## Data Availability

Not applicable.

## Supplementary Materials

The following supporting information can be downloaded at: https://www.mdpi.com/article/10.3390/ijerph192113869/s1. References [52,53] are cited in the Supplementary Materials.

## References

[1] Guo J., Zhou Y., Wang Y., Chen Y., Zhang B., Zhang J. (2022). Methylsiloxanes risk assessment combining external and internal exposure for college students. Sci. Total Environ..

[2] Horii Y., Kannan K. (2008). Survey of organosilicones compounds, including cyclic and linear siloxanes, in personal-care and household products. Arch. Environ. Contam. Toxicol..

[3] (2011). Siliconces Environmental, Health, and Safety Center (SEHSC). http://sehsc.americanchemistry.com.

[4] Mojsiewicz-Pieńkowska K., Krenczkowska D. (2018). Evolution of consciousness of exposure to siloxanes-review of publications. Chemosphere.

[5] U.S. Environmental Protection Agency (U.S. EPA) (2007). United States High Production Volume Challenge Program List. https://iaspub.epa.gov/sor_internet/registry/substreg/list/details.do?listId=74.

[6] OECD (Organisation for Economic Co-operation and Development) (2007). Manual for the Assessment of Chemicals. http://www.oecd.org/env/ehs/risk-assessment/manualfortheassessmentofchemicals.htm.

[7] European Chemicals Agency (ECHA) (2015). UK Proposes Restriction on Octamethylcyclotetrasiloxane (D4) and Decamethylcyclopentasiloxane (D5) in Personal Care Products That Are Washed off in Normal Use.

[8] European Chemicals Agency (ECHA) (2016). Background Document to the Opinion on the Annex XV Dossier Proposing Restrictions on Octamethylcyclotetrasiloxane (D4) and Decamethylcyclopentasiloxane (D5).

[9] Wang X., Jasmin S., Jones K.C., Ping G. (2018). Occurrence and spatial distribution of neutral perfluoroalkyl substances and cyclic volatile methylsiloxanes in the atmosphere of the Tibetan Plateau. Atmos. Chem. Phys..

[10] Xu L., Shi Y., Liu N., Cai Y. (2015). Methyl siloxanes in environmental matrices and human plasma/fat from both general industries and residential areas in China. Sci. Total Environ..

[11] Buser A.M., Kierkegaard A., Bogdal C., MacLeod M., Scheringer M., Hungerbühler K. (2013). Concentrations in ambient air and emissions of cyclic volatile methylsiloxanes in Zurich, Switzerland. Environ. Sci. Technol..

[12] Guo J., Zhou Y., Cui J., Zhang B., Zhang J. (2019). Assessment of volatile methylsiloxanes in environmental matrices and human plasma. Sci. Total Environ..

[13] Guo J., Zhou Y., Sun M., Cui J., Zhang B., Zhang J. (2020). Methylsiloxanes in plasma from potentially exposed populations and an assessment of the associated inhalation exposure risk. Environ. Int..

[14] Sparham C., Van Egmond R., O’Connor S., Hastie C., Whelan M., Kanda R. (2008). Determination of decamethylcyclopentasiloxane in river water and final effluent by headspace gas chromatography/mass spectrometry. J. Chromatogr. A.

[15] Zhang Z., Qi H., Ren N., Li Y., Gao D., Kannan K. (2011). Survey of cyclic and linear siloxanes in sediment from Songhua river and in sewage sludge from wastewater treatment plants, Northeastern China. Arch. Environ. Contam. Toxicol..

[16] Guo J., Zhou Y., Zhang B., Zhang J. (2019). Distribution and evaluation of the fate of cyclic volatile methyl siloxanes in the largest lake of southwest China. Sci. Total Environ..

[17] Horii Y., Minomo K., Ohtsuka N., Motegi M., Nojiri K., Kannan K. (2017). Distribution characteristics of volatile methylsiloxanes in Tokyo Bay watershed in Japan: Analysis of surface waters by purge and trap method. Sci. Total Environ..

[18] Sanchez-Brunete C., Miguel E., Albero B., Tadeo J.L. (2010). Determination of cyclic and linear siloxanes in soil samples by ultrasonic-assisted extraction and gas chromatography-mass spectrometry. J. Chromatogr. A.

[19] Sanchis J., Cabrerizo A., Galban-Malagon C., Barcelo D., Farre M., Dachs J. (2015). Unexpected occurrence of volatile dimethylsiloxanes in Antarctic soils, vegetation, phytoplankton, and krill. Environ. Sci. Technol..

[20] Sanchís J., Martínez E., Ginebreda A., Farré M., Barceló D. (2013). Occurrence of linear and cyclic volatile methylsiloxanes in wastewater, surface water and sediments from Catalonia. Sci. Total Environ..

[21] Lee D., Park M.-K., Lee I.-S., Choi S.-D. (2019). Contamination characteristics of siloxanes in coastal sediment collected from industrialized bays in South Korea. Ecotox. Environ. Saf..

[22] Powell D.E., Schoyen M., Oxnevad S., Gerhards R., Bohmer T., Koerner M. (2018). Bioaccumulation and trophic transfer of cyclic volatile methylsiloxanes (cVMS) in the aquatic marine food webs of the Oslofjord, Norway. Sci. Total Environ..

[23] Hanssen H., Warner N.A., Braathen T., Odland J.Ø., Lund E., Nieboer E., Sandanger T.M. (2013). Plasma concentrations of cyclic volatile methylsiloxanes (cVMS) in pregnant and postmenopausal Norwegian women and self-reported use of personal care products (PCPs). Environ. Int..

[24] Wang D.G., de Solla S.R., Lebeuf M., Bisbicos T., Barrett G.C., Alaee M. (2017). Determination of linear and cyclic volatile methylsiloxanes in blood of turtles, cormorants, and seals from Canada. Sci. Total Environ..

[25] Guo J., Zhou Y., Wang Y., Zhang B., Zhang J. (2021). Assessment of internal exposure to methylsiloxanes in children and associated non-dietary exposure risk. Environ. Int..

[26] Environment Canada, Health Canada (2008). Screening Assessment for the Challenge: Decamethylcyclopentasiloxane (D5). http://ec.gc.ca/ese-ees/default.asp?lang=En&n=13CC261E-5FB0-4D33-8000.

[27] Brooke D.N., Crookes M.J., Gray D., Robertson S. (2009). Environmental Risk Assessment Report: Octamethylcyclotetrasiloxane.

[28] Brooke D.N., Crookes M.J., Gray D., Robertson S. (2009). Environmental Risk Assessment Report: Decamethylcyclopentasiloxane.

[29] Brooke D.N., Crookes M.J., Gray D., Robertson S. (2009). Environmental Risk Assessment Report: Dodecamethylcyclohexasiloxane.

[30] Quinn A.L., Regan J.M., Tobin J.M., Marinik B.J., McMahon J.M., McNett D.A., Sushynski C.M., Crofoot S.D., Jean P.A., Plotzke K.P. (2007). In vitro and in vivo evaluation of the estrogenic, androgenic, and progestagenic potential of two cyclic siloxanes. Toxicol. Sci..

[31] McKim J.M., Wilga P.C., Breslin W.J., Plotzke K.P., Gallavan R.H., Meeks R.G. (2001). Potential estrogenic and antiestrogenic activity of the cyclic siloxane octamethylcyclotetrasiloxane (D4) and the linear siloxane hexamethyldisiloxane (HMDS) in immature rats using the uterotrophic assay. Toxicol. Sci..

[32] Meeks R.G., Stump D.G., Siddiqui W.H., Holson J.F., Plotzke K.P., Reynolds V.L. (2007). An inhalation reproductive toxicity study of octamethylcyclotetrasiloxane (D4) in female rats using multiple and single day exposure regimens. Reprod. Toxicol..

[33] Granchi D., Cavedagna D., Ciapetti G., Stea S., Schiavon P., Giuliani R., Pizzoferrato A. (1995). Silicone breast implants: The role of immune system on capsular contracture formation. J. Biomed. Mater. Res..

[34] Mackay D., Di Guardo A., Paterson S., Kicsi G., Cowan C.E. (1996). Assessing the fate of new and existing chemicals: A five-stage process. Environ. Toxicol. Chem..

[35] Mackay D., Di Guardo A., Paterson S., Cowan C.E. (1996). Evaluating the environmental fate of a variety of types of chemicals using the EQC model. Environ. Toxicol. Chem..

[36] Whelan M.J. (2013). Evaluating the fate and behaviour of cyclic volatile methyl siloxanes in two contrasting North American lakes using a multi-media model. Chemosphere.

[37] Mackay D., Hughes L., Powell D.E., Kim J. (2014). An updated Quantitative Water Air Sediment Interaction (QWASI) model for evaluating chemical fate and input parameter sensitivities in aquatic systems: Application to D5 (decamethylcyclopentasiloxane) and PCB-180 in two lakes. Chemosphere.

[38] Xu L., Shi Y., Cai Y. (2013). Occurrence and fate of volatile siloxanes in a municipal Wastewater Treatment Plant of Beijing, China. Water Res..

[39] (2017). Beijing Municipal Bureau of Statistics (BMBS). http://tjj.beijing.gov.cn/tjsj/yjdsj/rk/2017/201801/t20180119_391224.html.

[40] Genualdi S., Harner T., Cheng Y. (2011). Global distribution of linear and cyclic volatile methyl siloxanes in air. Environ. Sci. Technol..

[41] Yucuis R.A., Stanier C.O., Hornbucke K.C. (2013). Cyclic siloxanes in air, including identification of high levels in Chicago and distinct diurnal variation. Chemosphere.

[42] Kierkegaard A., McLachlan M.S. (2013). Determination of linear and cyclic volatile methylsiloxanes in air at a regional background site in Sweden. Atmos. Environ..

[43] Krogseth I.S., Kierkegaard A., McLachlan M.S., Breivik K., Hansen K.M., Schlabach M. (2013). Occurrence and seasonality of cyclic volatile methyl siloxanes in Arctic air. Environ. Sci. Technol..

[44] Kaj L., Schlabach M., Andersson J., Palm C.A., Schmidbauer N., Brorström-Lundén E. (2005). Siloxanes in the Nordic Environment.

[45] Lu Y., Yuan T., Wang W.H., Kannan K. (2011). Concentrations and assessment of exposure to siloxanes and synthetic musks in personal care products from China. Environ. Pollut..

[46] Companioni-Damas E.Y., Santos F.J., Galceran M.T. (2012). Analysis of linear and cyclic methylsiloxanes in sewage sludges and urban solls by concurrent solvent recondensation-large volume injection-gas chromatography-mass spectrometry. J. Chromatogr. A.

[47] Kierkegaard A., Van Egmond R., McLachlan M.S. (2011). Cyclic Volatile Methylsiloxane Bioaccumulation in Flounder and Ragworm in the Humber Estuary. Environ. Sci. Technol..

[48] Sparham C., Van Egmond R., Hastie C. (2011). Determination of decamethylcyclopentasiloxane in river and estuarine sediments in the UK. J. Chromatogr. A.

[49] Powell D.E., Kozerski G.E. (2007). Cyclic methylsiloxane (cVMS) materials in surface sediments and cores for Lake Ontario. HES Study No. 10724-108. Health and Environmental Sciences, Dow Corning Corporation, Auburn, Michigan: Study Submitted to Centre Europ6en des Silicones(CES).

[50] European Chemicals Agency (ECHA) (2018). Annex XVII to Regulation (EC) No 1907/2006 of the European Parliament and Council concerning the Registration, Evaluation, Authorization and Restriction of Chemicals (REACH) With Regard to Octamethylcyclotetrasiloxane (D4) and Decamethylcyclopentasiloxane (D5).

[51] Horii Y., Kannan K. (2019). Main Uses and Environmental Emissions of Volatile Methylsiloxanes. Volatile Methylsiloxanes in the Environment.

[52] Kala S.V., Lykissa E.D., Lebovitz R.M. (1997). Detection and Characterization of Poly(dimethylsiloxane)s in Biological Tissues by GC/AED and GC/MS. Anal. Chem..

[53] Xu S., Kozerski G.E. (2007). Assessment of the Fundamental Partitioning Properties of Permethylated Cyclosiloxanes.

