# Supplementary material for: Occurrence and Behavior of Methylsiloxanes in Urban Environment in Four Cities of China
Source: Int J Environ Res Public Health. 2022 Oct 25;19(21):13869. doi: 10.3390/ijerph192113869 (PMC9658807; doi:10.3390/ijerph192113869)
Supplement: Supplementary file 1 [file ijerph-19-13869-s001.zip › ijerph-1932578-supplementary.pdf]

SUPPORTING INFORMATION

Occurrence and Behaviour of Methylsiloxanes in Urban Envi-ronment in four cities of China

Yao Jiang [a], Junyu Guo [a*], Ying Zhou [b*], Boya Zhang [c], Jianbo Zhang [b]

[a]*College of Life and Environmental Sciences, Minzu University of China, Beijing 100081, China*

[b] *State Key Joint Laboratory for Environmental Simulation and Pollution Control, College of Environmental Sciences and Engineering, Peking University, Beijing, 100871, China*

[c] *Department of Epidemiology, University of Michigan, Ann Arbor, MI, USA*

** Correspondence: jyguo16@ pku.edu.cn (J. Guo); zhou.ying@pku.edu.cn (Y. Zhou)*

Number of pages: 19

Number of tables: 9

Number of figures: 2

Contents

S1 Text sections 3
S1.1 Chemicals 3
S1.2 Sample collection 3
S1.3 Extraction and analysis 4
S1.3.1 Air sample 4
S1.3.2 Water sample 4
S1.3.3 Soil sample 4
S1.3.4 Sediment sample 5
S1.3.5 GC-MS analysis 5
S1.4 Quality assurance/quality control (QA/QC) 5
S1.4.1 Air sample 5
S1.4.2 Water sample 6
S1.4.3 Soil sample 6
S1.4.4 Sediment sample 7
S1.5 Modeling assessment 7
S2 Table 10
S3 Figure 17
References 19

S1 Text sections

S1.1 Chemicals

Cyclic MSs (D4, D5, and D6, purity > 98%), polydimethylsiloxane mixture (PDMS), linear MSs (LMSs; L5–L16, purity > 98%), and tetrakis (trimethylsilyloxy)-silane (M4Q, purity 97%) standards were purchased from Sigma-Aldrich (St. Louis, MO, USA). The compositions of linear methylsiloxanes (LMSs, L5–L16) in the PDMS standard were measured by gas chromatography coupled with atomic emission detection (GC/AED) according to the previous studies (Kala et al., 1997; Xu et al., 2015), and similar results were obtained (Table S1). $^{13}C_8$-D4 (98% purity), $^{13}C_{10}$-D5 (98% purity), and $^{13}C_6$-D6 (98% purity) were purchased from Cambridge Isotope Laboratories (Tewksbury, MA, USA). ^{13}C-labeled CMSs and M4Q were used as internal standards for CMSs and LMSs, respectively. Methanol, n-hexane, ethyl acetate, and dichloromethane were purchased from Fisher Scientific (Fair Lawn, New Jersey, USA).

S1.2 Sample collection

ISOLUTE ENV+ 200 mg cartridges (Biotage AB, Uppsala, Sweden) were used to collect indoor air samples. The cartridges were connected to a low-flow-rate air sampler (KB-6120; Qingdao Jingcheng, Inc., Qingdao, China). Each air sample was collected for 24 h with sampling rates of 2 L/min. After sampling, the cartridges were capped and stored in a screw-top amber glass bottle plugged with stoppers to prevent contact with ambient air. All air samples were stored at −20°C. River water samples were collected by immersing a PTFE bottle under the water surface. All sample bottles were filled to the top, with no head space. After sample, all water samples were sealed and stored at 4°C. Each soil sample (~5 g) was collected using a steel syringe and quickly ejected into glass containers without headspace. All soil samples were stored at −20°C until analysis. Sediment samples were collected at each site using a BEEKER sediment sampler. Sample was collected from the upper 1 cm of the sediment and transferred to 1 L PTFE sample bottles. After sample, all samples were sealed and stored at −20°C. All samples were analyzed immediately after transportation to the laboratory. All samples (plasma, air, dust and PCPs) were treated within 4 days after sampling in this study.

S1.3 Extraction and analysis

S1.3.1 Air sample

Prior to extraction, 50 μL of internal standards ($^{13}C_8$-D4, $^{13}C_{10}$-D5, $^{13}C_6$-D6 and M4Q, 1 mg/L) were respectively added to the ENV+ cartridge, which was immediately eluted with 10 mL of n-hexane. The eluates were concentrated to 1 mL by a gentle stream of nitrogen and transferred into a GC vial for gas chromatography-mass spectrometry (GC-MS) analysis.

S1.3.2 Water sample

Firstly, the C18 SPE cartridges (300mg, Anple Inc., China) were conditioned with 6 mL of dichloromethane and 6 mL of methanol (flow rate of 1 mL/min) to enables the wetting of the packing material and the solvation of the functional groups. In addition, it removes possible impurities initially contained in the sorbent or the packaging. Secondly, 50 μL of internal standards ($^{13}C_8$-D4, $^{13}C_{10}$-D5, $^{13}C_6$-D6 and M4Q, 1 mg/L) was added in 500 mL of water sample (pH adjusted to 7 with HCl 1M). Then, the water samples were passed through the cartridges (flow rate of 10 mL/min) using 12 Position Vacuum Manifold Set-Complete (Anple Inc., China). During this step, the CMSs are concentrated on the sorbent. Thirdly, the elution of the CMSs by 6mL of dichloromethane (flow rate of 1 mL/min). Care must be taken not to allow the solid sorbent to dry between the conditioning and the sample treatment steps. Finally, the eluate was dried using a column containing anhydrous Na_2SO_4 and concentrated under a gentle stream of nitrogen. Then, Transfer concentrated extracts to a 1-mL volumetric flask, dilute with n-hexane to exactly 1 mL and transferred into a GC vial for gas chromatography-mass spectrometry (GC-MS) analysis.

S1.3.3 Soil sample

Soil samples were sieved through a 500 μm mesh sieve. 0.2 g of the sieved sample was spiked with 100 μL of internal standards ($^{13}C_8$-D4, $^{13}C_{10}$-D5, $^{13}C_6$-D6 and M4Q, 1 mg/L). The spiked sample was vortexed for 5 min at 2500 rpm with 10 mL of ethyl acetate/n-hexane mixture (1:1), and extracted by ultrasonic for 15 min with low temperature (0 ℃). Then, the mixture was centrifuged at 3500 rpm for 10 min, and the supernatant was transferred to a glass tube. Each sample was extracted three times. The total extract was concentrated to 2 mL with a gentle stream of nitrogen, and dried by a cartridge filled with 1.0 g of sodium sulfate. The cartridge was eluted with 5 mL of ethyl acetate/n-hexane mixture (1:1). The eluent was collected in a 10 mL glass tube

and was concentrated to 1 mL by a gentle stream of nitrogen and transferred into a GC vial for gas chromatography-mass spectrometry (GC-MS) analysis.

S1.3.4 Sediment sample

Sediment samples were freeze-dried with a freeze dryer, ground, and then passed through a 42-mesh sieve. After sieving, each sediment sample has been extracted immediately. 1 g of sample was placed in a centrifuge tube with the addition of 100 μL of internal standards ($^{13}C_8$-D4, $^{13}C_{10}$-D5, $^{13}C_6$-D6 and M4Q, 1 mg/L) and ethyl acetate/n-hexane mixed solvent (volume ratio 1:1). The mixture was sonicated for 20 min and centrifuged at 3500 rpm for 10 min. The solution was extracted and the extract was transferred to a glass test tube. Sample extraction was repeated three times, and extraction solvent volume were 10, 10, and 5 mL, respectively. Then, the extracts were combined and concentrated to 1 mL under nitrogen gas. The concentrated extract was then purified by passing through a neutral silica gel column, eluting with 10 mL of dichloromethane/n-hexane mixed solvent (volume ratio 1:4), and the purified solution were concentrated under a gentle stream of nitrogen. Then, Transfer concentrated extracts to a 1-mL volumetric flask, dilute with n-hexane to exactly 1 mL and transferred into a GC vial for gas chromatography-mass spectrometry (GC-MS) analysis.

S1.3.5 GC-MS analysis

Samples were analyzed using a GC-MS system (QP2010 SE; Shimadzu, Kyoto, Japan). The samples were separated on a 30-m DB5-MS column (0.25-mm id, 0.25-μm film thickness; J&W Scientific, Folsom, CA, USA) with a constant flow rate of helium gas (1.0 mL/min). MS was performed with an electron impact (EI) ion source and a selected ion monitoring (SIM) mode was used for detection. A low-bleed septum (Shimadzu, Kyoto, Japan) was used in the injector and the injection volume was 2 μL. The injector port (splitless mode), ion source, mass analyzer, and interface temperatures for D4–D6 were set to 200°C, 230°C, 150°C, and 280°C, respectively. For L5–L16, the corresponding temperatures were 300°C, 280°C, 150°C, and 280°C. The quantifier and qualifier ions used to monitor the signal of each compound are shown in Table S2.

S1.4 Quality assurance/quality control (QA/QC)

S1.4.1 Air sample

For air samples, limits of quantitation (LOQs) of methylsiloxanes (D4–D6 and L5–L16) ranged from 0.19–0.37 ng/m^3 for 24 h. The target compounds were added to the

ENV+ cartridges at four spiked concentrations levels (10, 100, 1000, and 10000 ng/m^3) (spiked blanks), and were extracted as real air samples before detection as mentioned in Section S1.3.2. The recoveries were between 84% and 93%, and their peak area relative standard deviations (RSD, %) (n = 3) ranged from 3.7–8.5%. The recoveries of internal standards $^{13}C_8$-D4, $^{13}C_{10}$-D5, $^{13}C_6$-D6 and M4Q are 89 ± 5.0%, 91 ± 4.2%, 90 ± 5.9%, and 90 ± 4.8%, respectively. D4, D5, and D6 were detected in procedural blanks at trace levels in air samples (0.08–0.11 ng/m^3), while LMSs (L5–L16) were not observed in procedural blanks. Parallel samples were collected at the same site to assess the repeatability of air sampling. The RSD of MSs concentrations in parallel air samples (n = 3) were less than 15%. To monitor potential losses during the sampling and storage period, spiked filed blanks (50 and 5000 ng/m^3) were stored and then analyzed as samples. The recoveries were between 93–97% and 94–97%, and their peak area RSD (n = 3) ranged from 3.5–5.6% and 3.0–4.9%. Blank cartridges was used for the field blanks, which were treated with the same procedure as the actual samples, except that no air was introduced to the cartridges.

S1.4.2 Water sample

Limits of quantitation (LOQs) of methylsiloxanes (D4–D6 and L5–L16) ranged from 9.0–18.3 ng/L. At three spiked concentrations levels (10, 100, and 1000 ng/g), recoveries of the seventeen compounds ranged from 85% to 90%, and their peak area relative standard deviations (RSD, %) (n = 3) ranged from 4.6–9.1%. The recoveries of internal standards $^{13}C_8$-D4, $^{13}C_{10}$-D5, $^{13}C_6$-D6 and M4Q are 85 ± 5.7%, 88 ± 6.3%, 86 ± 7.0%, and 88 ± 4.5%. D4, D5, and D6 were detected in procedural blanks at trace levels in water samples (1.0–2.1 ng/L), while LMSs (L5–L16) were not observed in procedural blanks. Ultrapure water was used for the field blanks, which were stored in polytetrafluoroethylene (PTFE) sample bottles and brought to the sampling site. The bottle mouth was opened during the sampling process, and the blanks were brought back to the laboratory with the samples.

S1.4.3 Soil sample

Limits of quantitation (LOQs) of methylsiloxanes (D4–D6 and L5–L16) ranged from 0.5–0.8 ng/g dw. At three spiked concentrations levels (2, 200, and 1000 ng/g), recoveries of the seventeen compounds ranged from 82% to 90%, and their peak area relative standard deviations (RSD, %) (n = 3) ranged from 5.0–8.8%. The recoveries of internal standards $^{13}C_8$-D4, $^{13}C_{10}$-D5, $^{13}C_6$-D6 and M4Q are 86 ± 6.1%, 89 ± 5.4%, 85

± 4.6%, and 89 ± 4.2%. D4, D5, and D6 were detected in procedural blanks at trace levels in soil samples (0.2–0.6 ng/g), while LMSs (L5–L16) were not observed in procedural blanks. Field blank of soil samples were collected from the reservoir that located in the remote area of southwestern China. Then, soil samples were treated according to a method described previously (Companioni-Damas et al., 2012). Sediment samples were thermally treated for five days at 50 ℃ to remove the target compounds. After this treatment, an appropriate amount of water was added to this material. Since the concentrations of MSs in this sample were lower than the limits of detection, there were used as a field blank. The field blanks were stored in PTFE bottles and brought to the sampling site. During the sampling process, the bottle mouth was opened. After sampling, the field blanks were brought back to the laboratory with other samples.

S1.4.4 Sediment sample

Limits of quantitation (LOQs) of methylsiloxanes (D4–D6 and L5–L16) ranged from 0.6–0.8 ng/g dw. At three spiked concentrations levels (2, 200, and 1000 ng/g), recoveries of the seventeen compounds ranged from 85% to 91%, and their peak area relative standard deviations (RSD, %) (n = 3) ranged from 4.7–9.2%. The recoveries of internal standards $^{13}C_8$-D4, $^{13}C_{10}$-D5, $^{13}C_6$-D6 and M4Q are 85 ± 4.6%, 88 ± 5.1%, 87 ± 6.0%, and 90 ± 5.5%. D4, D5, and D6 were detected in procedural blanks at trace levels in soil samples (0.2–0.6 ng/g), while LMSs (L5–L16) were not observed in procedural blanks. Field blank of sediment samples were same as blank of soil samples.

S1.5 Modeling assessment

Partition coefficients were temperature-adjusted using the Arrhenius equation and applying the activation energies. The temperature dependence of chemical partitioning between phases A and B can be represented by

$$K_{AB}(T) = K_{AB}(T_r) \cdot exp\left(\frac{\Delta U_{AB}}{R} \cdot \left(\frac{1}{T_r} - \frac{1}{T}\right)\right), \quad \text{(S1)}$$

where $K_{AB}(T)$ is the partition coefficient at the actual temperature of the lake (T, K), $K_{AB}(T_r)$ is the partition coefficient at the reference temperature (Tr, 298 K), □ΔU_{AB} is the energy of phase transfer (J·mol^{-1}) and R is the gas constant (J·mol^{-1}·K^{-1}).

Based on the assumption of first order kinetics, the relationship between the

degradation rate constant (K) and the half-life (τ_r) in the aquatic environment can be written as

$$k(T_r) = \frac{ln(2)}{\tau_{r,W}}, \tag{S2}$$

and the Arrhenius equation is

$$k(T) = k(T_r) \cdot exp\left(\frac{E_a}{R} \cdot \left(\frac{1}{T_r} - \frac{1}{T}\right)\right), \tag{S3}$$

where $k(T)$ is the rate constant at the actual temperature of the lake (T, K), $k(T_r)$ is the rate constant at the reference temperature (Tr, 298 K), E_a is the activation energy (kJ·mol^{-1}) (87.6, 87.2 and 93.5 for D4, D5 and D6, respectively (Xu and Kozerski, 2007) and R is the gas constant (J·mol^{-1}·K^{-1}).

S2 Table

Table S1. The compositions (%) of linear methylsiloxanes (L5–L16) in the PDMS standard.

	L5	L6	L7	L8	L9	L10	L11	L12	L13	L14	L15	L16
(%)	0.98	7.06	17.74	18.37	14.51	11.30	9.24	6.68	5.35	4.11	2.92	1.77

Table S2. Quantifier and qualifier ions of each compounds analysed by GC-MS.

Compounds	Abbreviation	Quantifier Ions (m/z)	Qualifier Ions (m/z)
Octamethylcyclotetrasiloxane	D4	281	133, 265
Decamethylcyclopentasiloxane	D5	267	73, 355
Dodecamethylcyclohexasiloxane	D6	429	73, 341
Dodecamethylpentasiloxane	L5	221	281, 369
Tetradecamethylhexasiloxane	L6	221	281, 369
Hexadecamethylheptasiloxane	L7	221	281, 369
Octadecamethylnonasiloxane	L8	221	281, 369
Eicosamethylnonasiloxane	L9	221	281, 369
Docosamethyldecasiloxane	L10	221	281, 369
Tetracosamethylundecasiloxane	L11	221	281, 369
Hexacosamethyldodecasiloxane	L12	221	281, 369
Octacosamethyltridecasiloxane	L13	221	281, 369
Triacontamethyltetradecasiloxane	L14	221	281, 369
Dotriacontmethylpentadecasiloxane	L15	221	281, 369
Tetraiacontamethylhexadecasiloxane	L16	221	281, 369
Octamethylcyclotetrasiloxane-$^{13}C_8$	^{13}C-D4	288	305, 333
Decamethylcyclopentasiloxane-$^{13}C_{10}$	^{13}C-D5	272	76, 364
Dodecamethylcyclohexasiloxane-$^{13}C_{12}$	^{13}C-D6	440	348, 459
tetrakis (trimethylsilyoxy) silane	M4Q	369	147, 281

Table S3. Properties of D4, D5 and D6 used as inputs to the EQC model

Property	D4	D5	D6	Source
Molar mass (g/mol)	297	371	445	[2]
Melting Point (°C)	17.5	−38	−3	[2]
Vapor Pressure (Pa)	122	30.4	2.2	[2]
Aqueous solubility (g/m^3)	0.056	0.017	0.0053	[2]
Henry's Law Constant (Pa/m^3·mol)	1220000	3340000	4950000	[2]
$\log K_{OW}$	6.98	8.07	8.87	[3]
$\log K_{AW}$	2.69	3.13	3.30	[3]
$\log K_{OC}$	4.22	5.20	6.03	[3]
ΔU_{OW} (kJ/mol)	7.9	29	33.6	[4]
ΔU_{AW} (kJ/mol)	51.9	80.4	92.1	[4]
ΔU_{OC} (kJ/mol)	7.9	29	33.6	[4]
Half-life in air at 25°C (h)	108	101	79	[5]
Half-life in water at 25°C (h)	9.6	216	960	[6]
Half-life in soil at 25°C (h)	127	302	9624	[5]
Half-life in sediment at 25°C (h)	8760	74400	140055	[5]

Table S4. The emission rates of CMSs (D4–D6) for atmosphere, water and soil

Chemical	Air (kg/h)	Water (kg/h)	Soil (kg/h)
D4	13.9	0.26	1.9×10^{-2}
D5	17.1	0.43	2.9×10^{-2}
D6	12.7	0.40	2.2×10^{-2}

Table S5. The environmental parameters of Beijing

Environmental media	Area (m^2)	Height/Depth (m)	Density (kg/m^3)
Air	1.6×10^{10}	1000	-
Water	3.0×10^{8}	10	-
Soil	1.0×10^{10}	0.1	1500
Sediment	3.0×10^{8}	0.05	1280

Table S6. The actual value (%) of the fraction of the total mass of CMSs (D4–D6) in the each environmental media in Beijing

Chemical	Air	Water	Soil	Sediment
D4	92.6	2.2	0	5.2
D5	8.2	1.5	3.3	87.0
D6	3.4	0.91	2.7	92.9

Table S7. The standard deviation of the mean concentrations (ng/m^3) of methylsiloxanes in air samples from four cities.

**Site**	**D4**	**D5**	**D6**	**ΣL5-L8**
Beijing (urban)	2.8	4.1	2.5	<LOD [1]
Beijing (suburban)	3.6	4.0	6.1	<LOD
Kunming (urban)	3.3	2.9	3.7	<LOD
Kunming (suburban)	5.1	3.5	4.2	<LOD
Lijiang (urban)	4.4	6.3	2.6	<LOD
Lijiang (suburban)	5.7	3.2	3.8	<LOD
Zhangjiagang (urban)	2.6	4.5	6.0	<LOD
Zhangjiagang (suburban)	3.1	6.2	5.7	<LOD
Siloxane production plan (around)	9.2×10^{3}	7.8×10^{3}	8.5×10^{3}	9.9
Siloxane production plan (downwind)	2.5×10^{3}	1.9×10^{3}	7.3×10^{2}	6.4
Siloxane production plan (upwind)	6.3×10^{2}	3.6×10^{2}	11	<LOD

[1] <LOD = levels lower than LOD.

Table S8. The standard deviation of the mean concentrations (ng/L) of methylsiloxanes in water samples from four cities.

City	Site	D4	D5	D6	ΣL3-L5
Beijing	River a (1)	<LOD [1]	2.6	<LOD	<LOD
	River a (2)	<LOD	2.2	<LOD	<LOD
	River b (1)	<LOD	4.1	<LOD	<LOD
	River b (2)	<LOD	3.5	<LOD	<LOD
	River c (1)	<LOD	2.9	2.0	<LOD
	River c (2)	<LOD	3.8	4.3	<LOD
	River d (1)	3.1	8.0	7.2	<LOD
	River d (2)	2.3	5.1	4.5	<LOD
	River e (1)	2.9	4.8	3.7	<LOD
	River e (2)	1.8	3.5	2.7	<LOD
	River f (1)	2.2	6.9	3.0	<LOD
	River f (2)	1.9	3.4	5.6	<LOD
Kunming	River g (1)	<LOD	2.1	<LOD	<LOD
	River g (2)	<LOD	2.3	<LOD	<LOD
	River h (1)	1.8	6.2	4.5	<LOD
	River h (2)	<LOD	3.1	2.4	<LOD
	River j (1)	2.6	4.0	2.8	<LOD
	River j (2)	<LOD	3.6	1.9	<LOD
Lijinag	River k (1)	<LOD	2.6	3.0	<LOD
	River k (2)	2.1	2.9	4.1	<LOD
	River m (1)	<LOD	<LOD	<LOD	<LOD
	River m (2)	<LOD	<LOD	<LOD	<LOD
	River n (1)	<LOD	<LOD	<LOD	<LOD
	River n (2)	<LOD	<LOD	<LOD	<LOD
Zhangjiagang	River p (1)	<LOD	3.4	2.7	<LOD
	River p (2)	<LOD	2.3	3.3	<LOD
	River q (1)	3.0	5.4	3.6	<LOD

	River q (2)	1.8	4.9	2.4	<LOD
	River r (1)	8.6	10	12	4.8
	River r (2)	2.2	4.3	3.6	<LOD

[1] <LOD = levels lower than LOD.

Table S9. The standard deviation of the mean concentrations (ng/g dw) of methylsiloxanes in soil and sediment samples from four cities.

City	Site	Soil				Sediment			
		D4	D5	D6	ΣL5-L11	D4	D5	D6	ΣL5-L11
Beijing	River a (1)	<LOD [1]	2.4	<LOD	<LOD	0.16	6.7	5.3	7.2
	River a (2)	<LOD	<LOD	<LOD	<LOD	0.15	5.9	7.4	4.5
	River b (1)	<LOD	<LOD	<LOD	<LOD	0.12	3.9	6.0	5.7
	River b (2)	<LOD	<LOD	<LOD	<LOD	0.15	4.4	2.6	5.1
	River c (1)	<LOD	<LOD	<LOD	<LOD	0.20	8.1	6.8	6.3
	River c (2)	<LOD	<LOD	<LOD	<LOD	0.17	5.6	8.8	7.0
	River d (1)	<LOD	0.20	0.17	<LOD	2.3	83.5	62.4	44.7
	River d (2)	<LOD	<LOD	<LOD	<LOD	0.31	7.8	9.0	8.4
	River e (1)	<LOD	0.12	0.15	<LOD	1.4	54.3	68.0	12.7
	River e (2)	<LOD	<LOD	<LOD	<LOD	0.26	10.5	9.3	11.0
	River f	<LOD	0.17	0.20	<LOD	2.4	48.9	71.1	14.2

	(1)								
	River f (2)	<LOD	<LOD	<LOD	<LOD	0.31	10.8	8.6	7.3
Kunming	River g (1)	<LOD	<LOD	<LOD	<LOD	0.22	5.0	8.2	9.5
	River g (2)	<LOD	<LOD	<LOD	<LOD	0.30	6.1	4.7	5.5
	River h (1)	<LOD	0.24	0.28	<LOD	3.2	42.2	65.1	12.6
	River h (2)	<LOD	<LOD	<LOD	<LOD	0.16	7.3	10.1	9.2
	River j (1)	<LOD	0.22	0.17	<LOD	1.8	48.5	55.9	14.0
	River j (2)	<LOD	<LOD	<LOD	<LOD	0.29	11.1	10.4	12.6
Lijinag	River k (1)	<LOD	<LOD	<LOD	<LOD	<LOD	<LOD	<LOD	<LOD
	River k (2)	<LOD	<LOD	<LOD	<LOD	<LOD	<LOD	<LOD	<LOD
	River m (1)	<LOD	<LOD	<LOD	<LOD	<LOD	<LOD	<LOD	<LOD
	River m (2)	<LOD	<LOD	<LOD	<LOD	<LOD	<LOD	<LOD	<LOD
	River n (1)	<LOD	<LOD	<LOD	<LOD	0.19	5.1	6.2	3.9
	River n (2)	<LOD	<LOD	<LOD	<LOD	0.23	5.7	4.8	4.3
Zhangjiagang	River p (1)	<LOD	<LOD	<LOD	<LOD	0.20	5.9	7.0	7.5

River p (2)	<LOD	<LOD	<LOD	<LOD	0.24	6.1	10.7	10.2
River q (1)	<LOD	<LOD	<LOD	<LOD	0.30	9.5	12.2	10.8
River q (2)	<LOD	<LOD	<LOD	<LOD	0.26	8.7	11.3	11.0
River r (1)	3.8	5.5	7.1	9.6	7.5	60.3	58.4	71.6
River r (2)	<LOD	<LOD	0.21	<LOD	0.44	12.0	15.1	11.7

S3 Figure

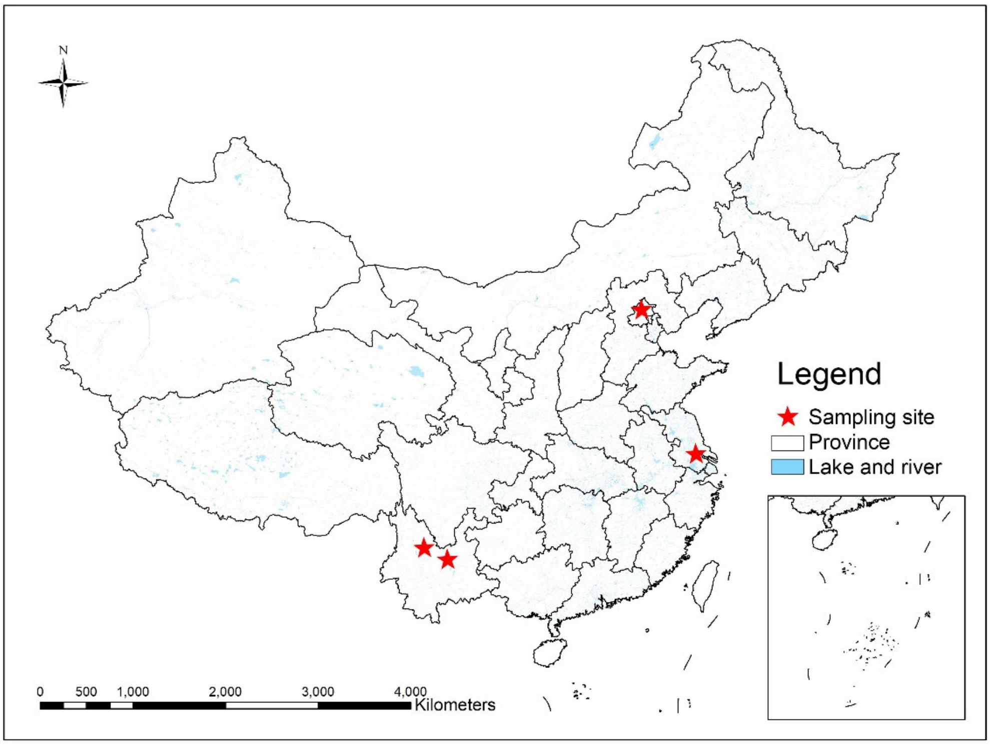

Figure S1. Sampling sites in four cities in China.

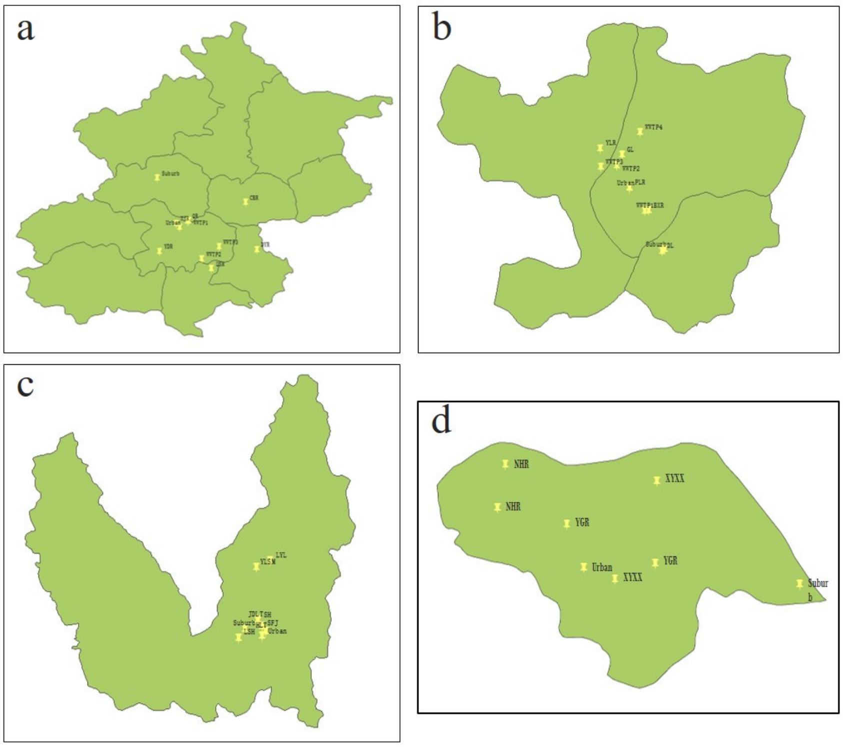

Figure S2. Local sampling site in Beijing (a), Kunming (b), Lijiang (c), and Zhangjiagang (d).

References

1. Kala, S. V., Lykissa, E. D., Lebovitz, RM., 1997. Detection and Characterization of Poly(dimethylsiloxane)s in Biological Tissues by GC/AED and GC/MS. Anal. Chem. 69, 1267-1272.

2. Xu, L., Shi, Y., Liu, N., Cai, Y., 2015. Methyl siloxanes in environmental matrices and human plasma/fat from both general industries and residential areas in China. Sci. Total Environ. 505, 454–463.

3. Companioni-Damas, E. Y., Santos, F. J., Galceran, M.T., 2012. Analysis of linear and cyclic methylsiloxanes in sewage sludges and urban soils by concurrent solvent recondensation – large volume injection – gas chromatography–mass spectrometry. J. Chromatogr. A. 1268, 150– 156.

4. Xu, S., Kozerski, G. E., 2007. Assessment of the fundamental partitioning properties of permethylated cyclosiloxanes. Poster presented at SETAC Europe, Porto, Portugal, May 2007.
